# Association between VNTR Polymorphism in Promoter Region of *Prodynorphin (PDYN)* Gene and Methamphetamine Dependence

**DOI:** 10.3889/oamjms.2015.079

**Published:** 2015-06-23

**Authors:** Khyber Saify, Mostafa Saadat

**Affiliations:** Department of Biology, College of Sciences, Shiraz University, Shiraz, Iran

**Keywords:** Methamphetamine dependence, *PDYN*, Polymorphism, VNTR, Susceptibility

## Abstract

**AIM:**

Prodynorphin (*PDYN*; OMIM: 131340) is the precursor of the dynorphin related peptides which plays an important role in drug abuse. Previous studies have been shown that the expression of *PDYN* is regulated by a genetic polymorphism of VNTR in the promoter region of the gene.

**MATERIALS AND METHODS:**

The present case-control study was performed on 52 (41 males, 11 females) methamphetamine dependence patients and 635 (525 males, 110 females) healthy blood donors frequency matched with the patients according to age and gender, as a control group was participated in the study.

**RESULTS:**

The genotypes of VNTR *PDYN* polymorphism were determined using PCR method. The *HL* (OR = 1.22, 95%CI: 0.67-2.20, P = 0.500) and *LL* (OR = 0.86, 95%CI: 0.28-2.57, P = 0.792) genotypes does not alter the risk of methamphetamine dependence, in comparison with the *HH* genotypes.

**CONCLUSION:**

The present study revealed no association between the VNTR polymorphism in the promoter region of the *PDYN* gene and methamphetamine dependence risk.

## Introduction

Prodynorphin (PDYN; OMIM: 131340) is the precursor of the dynorphin related peptides which plays an important role in several complex traits such as drug abuse [1]. It has been reported that the expression level of the *PDYN* gene is significantly increased by cocaine [2]. On the other hand, the *Pdyn* knockout mice showed increased explorative behavior in anxiety tests suggesting an anxiogenic role of peptides derived from PDYN [3]. The PDYN family of neuropeptides comprises six peptides of varying lengths that are formed from the precursor PDYN and which activate kappa-opioid receptors (KORs) located in the peripheral and central nervous systems. The functional genetic polymorphisms in the *PDYN* gene may affect the level of transcription, or response to environmental or internal stimuli. This would in turn result in downstream neurobiological and behavioral adaptations (e.g., proximally through dynorphins’ agonist actions on KORs). Therefore, genetic variations or epigenetic changes of the PDYN could underlie vulnerability to drug dependence [4, 5].

It is well established that within the core promoter region of the *PDYN*, a 68-bp sequence was found to occur as a polymorphic element, either singular or as tandemly repeated two, three, four, or five times [6, 7]. This genetic polymorphism has an important role in regulation of *PDYN* expression. The expression level of *PDYN* was decreased as a function of number of repeats [6, 8].

Several studies investigated the association between the *PDYN* VNTR polymorphism and susceptibility to heroin, cocaine and methamphetamine dependence [7, 9-13]. Dependence to drugs is a heterogeneous chronic multifactorial disease that has major medical, social, and economic complications. Identification of the genetic polymorphisms may increase our understanding of the disorders, help in the development of new treatments and advance personalized medicine [14].

However, there is only published study investigating the association between this polymorphism and the risk of methamphetamine dependence in Japanese population [13].

There is no study in Caucasian populations. Therefore, the present case-control study was carried out. The aim of the present study is to investigate the association between *PDYN* VNTR polymorphism and methamphetamine dependence susceptibility.

## Materials and Methods

A detailed description of the study subjects has been reported in our recently published report [15]. Briefly, a total of 52 (41 males, 11 females) methamphetamine dependence patients and 635 (525 males, 110 females) healthy blood donors frequency matched with the patients according to age and gender, as a control group was participated in the study. The patients were in methadone maintenance for treating methamphetamine dependence and all of them reported methamphetamine as their primary drug of choice. The mean years (± SD) of duration of drugs used by the patients was 12.0 (± 8.1). There was no significant difference between the gender groups for this duration (t = 0.81, df = 50, P = 0.419). Control individuals were blood donors, who declared that they did not suffer substance abuse. Considering the high heterogeneity of the Iranian population [16-18], the participants were selected from Persian Muslims (Caucasians) living in Shiraz (Fars Province, southern Iran). Informed consent was obtained from each subject before the study, which was approved by the institutional review board of our university.

Peripheral blood samples were collected from the participants. Genomic DNA was isolated from EDTA treated blood samples. The PCR conditions for determining the genotypes of the *PDYN* VNTR polymorphism was the same as that reported previously [7]. The following primers were used: forward primer 5’-AGC AAT CAG AGG TTG AAG TTG GCA GC-3’ and reverse primer 5’-GCA CCA GGC GGT TAG GTA GAG TTG TC-3’. The PCR conditions consisted of an initial denaturation step of 94°C for 5min, followed by 30 cycles of 94°C for 30sec, 62°C for 45sec and 72°C for 45sec, a final extension of 72°C for 5min. The PCR products were subjected to 2.5% agarose gel electrophoresis and then visualized with a gel imaging system to determine the genotypes. Alleles containing 1, 2, 3, 4, or 5 repeats, produced PCR amplicons of 379, 447, 515, 583, and 651 bp, respectively. For quality control, 15% of randomly selected samples were repeated to verify genotyping results and 100% concordance was found. The alleles of the VNTR polymorphism were divided into two groups, L and H reflecting the relatively low expressed (1-2 repeats) and high expressed (3-5 repeats) alleles, respectively.

The associations between the genotypes of the study polymorphism and susceptibility to methamphetamine dependence were assessed by calculating odds ratios (ORs) and 95% confidence intervals (CIs). The reference group consisted of individuals with HH genotypes. Statistical analyses were performed using the SPSS version 11.5 statistical software package (SPSS Inc, Chicago, IL, USA). A probability of P<0.05 was considered statistically significant. All P values were two-tailed.

## Results and Discussion

Table 1 shows the genotype distribution of the VNTR polymorphism between the cases and controls. Considering that there was no significant difference between gender groups for the genotypes (For patients: χ^2^ = 0.53, df = 2, P = 0.764; For controls χ^2^ = 0.13, df = 2, P = 0.935), the gender groups were pooled. Table 2 shows the distribution of the genotypes according the L and H allele groups. The genotypic frequencies of the polymorphism in controls (χ^2^ = 0.61, df = 1, P = 0.432) and patients (χ^2^ = 0.36, df = 1, P = 0.547) were consistent with the Hardy-Weinberg equilibrium distribution.

**Table 1:** Genotypic distribution of *PDYN* VNTR polymorphism in control and methamphetamine dependence cases

Genotypes	Controls	Cases
**1/1**	1	0
**1/2**	5	0
**1/3**	5	0
**2/2**	55	4
**2/3**	242	24
**2/4**	11	0
**3/3**	292	21
**3/4**	22	2
**3/5**	1	1
**4/4**	1	0

**Table 2:** Association between *PDYN* VNTR polymorphism and risk of methamphetamine dependence

Genotypes/Alleles	Controls N (%)	Cases N (%)	OR	95% CI	P-Value
***HH***	316 (49.8)	24 (46.2)	1.0	-	-
***HL***	258 (40.6)	24 (46.2)	1.22	0.67-2.20	0.500
***LL***	61 (9.6)	4 (7.6)	0.86	0.28-2.57	0.792
**Alleles**					
***H***	890 (70.0)	72 (69.2)	1.0	-	-
***L***	380 (30.0)	32 (30.8)	1.04	0.67-1.61	0.856

Statistical analysis indicating that the HL (OR = 1.22, 95%CI: 0.67-2.20, P = 0.500) and LL (OR = 0.86, 95%CI: 0.28-2.57, P = 0.792) genotypes does not alter the risk of methamphetamine dependence, in comparison with the HH genotypes (Table 2). There is no significant interaction between gender and genotypes for alter the risk of dependency (P = 0.839). Also there is no association between allelic prevalence and methamphetamine dependence risk (OR=1.04, 95%CI: 0.67-1.61, P=0.856).

Few studies investigated the association between risk of heroin dependence and *PDYN* VNTR polymorphism with inconsistent results [6, 7, 9-13]. A significant association was reported between the *PDYN* VNTR polymorphism and methamphetamine dependence in Japan; alleles with three or four copies of the 68-bp repeat were found more frequently in Japanese individuals with methamphetamine dependence compared to healthy control group [13].

Association studies in complex disorders such as dependency to a drug are challenging. The inconsistency between our present study and previous study [13] investigating the association between *PDYN* VNTR polymorphism and risk of dependency to methamphetamine may be explained by several factors, including small sample size of the studies, inadequate statistics, ethnic heterogeneity and population stratification, large phenotype range, and different diagnostic criteria.

It should be mentioned that the main limitation of the present study is its sample size, especially drug abuse patient. Finally, considering the fact that ethnicity may influence the observed associations in multifactorial diseases [19], replication of this study in other countries is recommended.
